# Here to Stay: The Journey of “Junge Neuroradiologie” Has Only Just Begun

**DOI:** 10.1007/s00062-024-01387-2

**Published:** 2024-01-29

**Authors:** Daniel P. O. Kaiser

**Affiliations:** Faculty of Medicine and University Hospital, Carl Gustav Carus, Technische Universität Dresden, Fetscherstraße 74, 01307 Dresden, Germany

Reflecting on 3 years since its founding on 12 November 2020, the “Junge Neuroradiologie” (JUNRAD) remains dedicated to advocating for young neuroradiologists within the German Society of Neuroradiology (DGNR).

During this time, collaborating with leading German neuroradiologists and societies, JUNRAD has led numerous tailored initiatives to meet the specific needs of young neuroradiologists [1]. These efforts have significantly enriched the resources available to DGNR members and a broader community interested in neuroradiology. We have fostered change in line with the evolving landscape of our field, embracing modern technologies, especially in communication and education.

Notable initiatives such as “Meet the Expert”, “neuroradio:LOGISCH”, “Junge Neuroradiologie on demand”, “eFellowship Interventionelle Neuroradiologie”, “Fit für Neuroradiologie”, “Publikationspreis der Jungen Neuroradiologie”, “Raducation”, and efforts concerning the “Musterweiterbildungsordnung” have left a tangible impact on neuroradiology practices and policies within the DGNR, demonstrating clear progress and adaptability [2, 3].

With our newly elected team [4], JUNRAD remains steadfast in its commitment to the advancement of neuroradiology. We aim to further strengthen the promotion of young scientists, support access to existing research infrastructure, and facilitate connections between young scientists. Together with representatives of the German Medical Association, we will explore opportunities and requirements for improving regulations regarding the structured specialist training in neuroradiology. We strive to promote development and growth in our field.

Finally, heartfelt thanks go to Sarah Schläger, Vivien Richter, Isabelle Riederer, Katharina Schregel, Roland Schwab, Katharina Wenger-Alakmeh, Andrea Jarre, Katharina Fieseler, Saif Afat, Jonas Reis, Isabel Molwitz, Nina Keil-Wündisch, Florian Schneider, Claus Zimmer, Ansgar Berlis, Peter Schramm, the board members of the DGNR and Jennifer Linn, Martin Wiesmann, László Solymosi, and all the invaluable supporters of JUNRAD.
